# Perinatal phlorizin alleviates maternal high-fat diet-induced metabolic syndrome in female mouse offspring and is associated with modulation of the gut microbiota

**DOI:** 10.3389/fnut.2026.1799829

**Published:** 2026-06-10

**Authors:** Xueran Mei, Yi Yang, Yi Wen, Xiaoyu Zhang, Zhengjuan Li, Tai Yang, Liping Li

**Affiliations:** 1 Department of Obstetrics, Shanghai First Maternity and Infant Hospital, School of Medicine, Tongji University, Shanghai, China; 2 Department of Obstetrics and Gynecology, Tongji Hospital, Tongji Medical College, Huazhong University of Science and Technology, Wuhan, China; 3 Department of Pediatrics, Third Military Medical University Southwest Hospital, Chongqing, China; 4 College of Life Sciences, Sichuan Normal University, Chengdu, China; 5 Reproductive Medicine Center, Third School of Clinical Medicine, Peking University, Beijing, China; 6 School of Pharmacy, Chengdu Medical College, Chengdu, China

**Keywords:** female offspring, gut microbiota, metabolic syndrome, obesity susceptibility, phlorizin, transgenerational effects

## Abstract

**Introduction:**

Maternal obesity induces transgenerational metabolic syndrome (MS). The role of phlorizin (PHZ) in improving MS has been confirmed; however, the transgenerational metabolic benefits of PHZ in female offspring remain unclear. This study aimed to investigate whether perinatal PHZ intake could mitigate the adverse metabolic effects of maternal high-fat diet (HFD) in female offspring and to elucidate the role of the gut microbiota in mediating these transgenerational effects.

**Methods:**

C57BL/6 mice with maternal HFD ± perinatal PHZ (0.8 g/kg diet) intervention were used. After weaning, Female offspring’s glucolipid metabolism, gut barrier, gut microbiota, and SCFAs were analyzed. Obesogenic dietary challenge and fecal microbiota transplantation (FMT) were implemented to verify microbiota mediation.

**Results:**

Maternal HFD induces metabolic abnormalities in female offspring, characterized by disrupted glucolipid metabolism during weaning and mild obesity. In adulthood, although these offspring did not display overt obesity, they exhibited gut microbial dysbiosis (increase *Firmicutes/Bacteroidetes* ratio and pro-inflammatory bacteria), accompanied by insulin resistance and impaired intestinal barrier function, along with a significantly increased obesity susceptibility. Maternal PHZ co-intervention ameliorated MS and restored gut microbial balance in adulthood, increased the abundance of SCFA-producing bacteria (*Akkermansia muciniphila* and *Blautia* sp.), elevated fecal SCFAs and serum GLP1/2 levels, improved gut barrier integrity, alleviated inflammatory conditions, and reduced obesity susceptibility. To eliminate the protective effect of estrogen metabolism, antibiotic-treated (ABX) male mice were selected as the recipients for FMT. ABX male mice receiving FMT from PHZ-intervened female offspring could attenuate MS induced by receiving FMT from maternal HFD offspring via the gut microbiota–SCFA pathway.

**Conclusion:**

Our findings suggested that maternal PHZ alleviates maternal HFD-induced transgenerational metabolic dysfunction in female offspring and is associated with modulation of the gut microbiota, positioning PHZ as a promising functional food component with transgenerational metabolic protective potential.

## 1 Introduction

In recent decades, metabolic syndrome (MS) associated with obesity, such as insulin resistance, impaired glucose tolerance, and elevated body mass index, have emerged as a major global health challenge. According to the World Health Organization (WHO), in 2022, approximately one in eight individuals worldwide was affected by obesity, and 390 million children and adolescents aged 5 to 19 were overweight. It has been estimated that by the year 2050, more than half of adults and one-third of all children and adolescents will likely be affected by either overweight or obesity.^1^ Obesity and overweight pose significant threats to individuals’ lives by substantially increasing the risk of developing type 2 diabetes (T2D) mellitus, cardiovascular disease, stroke, atherosclerosis, and other life-threatening conditions, resulting in more than 5 million deaths annually due to related complications, according to the Global Burden of Diseases study.^2^ Based on a large body of observational studies (1–3) and animal experiments (4–6), the developmental origins of health and disease (DOHaD) hypothesis have been proposed. It posits that adverse exposures during critical windows in early life, including the prenatal period, can have long-term and irreversible effects later in life (7). A deleterious intrauterine environment resulting from maternal obesity may program offspring for increased risks of developing insulin resistance, glucose intolerance, altered lipid profiles, obesity, cardiovascular diseases, asthma, and even cancers in later adult life. A substantial body of evidence indicates that maternal high-fat diet during pregnancy and lactation increases the susceptibility to glucose intolerance, obesity, and other metabolic disorders in adult offspring (8–11). Therefore, pregnancy and lactation may represent critical windows during which disease trajectories can be reset.

Accumulating evidence has demonstrated an association between childhood obesity and gut microbiota in recent years. Gut dysbiosis leads to increased production of proinflammatory microbial metabolites, such as lipopolysaccharide (LPS), and disrupts the integrity of the intestinal barrier, thereby contributing to chronic systemic inflammation that sets the stage for the development of insulin resistance, T2D, and obesity (12, 13). What has challenged the ‘sterile womb’ paradigm for years is that advanced molecular techniques have detected microbes in the placenta, amniotic fluid and meconium (14–16), indicating that the in-utero environment is colonized by microbiota during prenatal development, thereby overturning a century-old dogma that the fetus develops in a germ-free (GF) environment within the uterus. One of the putative routes for bacterial access to the uterus is via the gastrointestinal tract. Maternal diets significantly influence the composition and diversity of the gut microbiota, thereby exerting indirect effects on the developing fetus. These effects may not only modulate infant metabolism but also persist into later life, with long-term health implications (17). Maternal obesity can lead to gut dysbiosis in the maternal gastrointestinal tract, and these compositional and metabolic disorders of gut microbiota can be transmitted to the infant, thereby increasing the risk of metabolic diseases in later life (18, 19). The study found that GF mice receiving fecal microbiota transplantation (FMT) from offspring born to obese mothers exhibited more severe obesity symptoms when fed a high-fat diet (20). Moreover, when gut microbiota from an obese and a lean identical twin were transplanted into GF mice, those receiving microbiota from the obese twin rapidly gained body weight even under a normal diet (21). Therefore, the gut microbiota plays an essential role in mediating the effects of maternal obesity and determining the development of obesity and other MS in offspring. This suggests that perinatal modulation of the gut microbiota may represent a promising therapeutic target for interrupting the transgenerational cycle of obesity from mother to child.

Phlorizin (PHZ) is a natural polyphenol belonging to the dihydrochalcone subclass of flavonoids, which was first identified from apple tree bark and is widely distributed in various fruits and plant species (22). Since its introduction as a pharmacological agent in physiological research over 150 years ago, PHZ has been shown to enhance insulin sensitivity and reduce blood glucose levels, leading to improvements in obesity- and diabetes-related symptoms in animal models. These effects are mediated through the inhibition of the sodium-glucose cotransporter expressed in the proximal renal tubules and the intestinal mucosa (22, 23). However, due to its extremely low bioavailability (less than 1%) (24), the gut microbiota may represent the primary site of action for its biological effects. Our previous research has demonstrated that PHZ can ameliorate HFD-induced MS via the “Gut microbiota–barrier” axis, with its therapeutic mechanism involving the modulation of gut microbiota and enhanced production of short-chain fatty acids (SCFAs) and intestinal peptide hormones (GLP-1/2) (25). Furthermore, PHZ significantly improved the gut microecology in diabetic (*db/db*) mice, as evidenced by a marked increase in *Akkermansia muciniphila* (SCFA-producing bacteria) bacteria and SCFAs, along with reduced serum LPS levels, ultimately contributing to the alleviation of T2D symptoms (26). However, studies exploring the effects and mechanism of perinatal phlorizin intake on transgenerational metabolic health are lacking.

Previous studies have predominantly focused on male offspring, which are more susceptible to maternal HFD-induced MS, while female offspring have long been understudied. This bias arises partly because estrogen exerts potent metabolic protective effects in premenopausal females, including enhancing insulin sensitivity, suppressing visceral fat accumulation, and reducing chronic inflammation, which complicates the phenotypic presentation of MS in female offspring (27). However, clinical and epidemiological evidence indicates that maternal obesity or HFD exposure still increases the long-term metabolic risk of female offspring, and the protective effect of estrogen gradually diminishes after menopause, leading to a sharp increase in the incidence of MS in postmenopausal women (28). Furthermore, the gut microbiota, a core mediator of transgenerational metabolic programming, exhibits significant sexual dimorphism, and maternal gut microbiota can be vertically transmitted to offspring with sex-specific differences (29). Therefore, systematically exploring the interventional effect and mechanism of PHZ on maternal HFD-induced metabolic disorders in female offspring is crucial to fill the research gap, clarify the complex mechanism of estrogen-gut microbiota interaction, and provide targeted nutritional intervention strategies for female offspring metabolic health.

In the current study, we aimed to investigate whether maternal PHZ administration during the perinatal period (pregnancy and lactation) could mitigate the adverse metabolic effects of maternal HFD in female offspring and influence their susceptibility to obesity in adulthood. Furthermore, we explored the potential role of the gut microbiota in mediating the transgenerational metabolic effects of maternal PHZ intake on female offspring. Overall, we found that (1) maternal HFD induces persistent metabolic abnormalities in female offspring. Although estrogen partially ameliorates the adult-onset obese phenotype, the offspring still suffer from impaired intestinal microecology, intestinal barrier and insulin function, and a significantly increased susceptibility to obesity; (2) maternal PHZ significantly improve the intestinal microecology of female offspring, including increasing the SCFAs-producing bacteria and intestinal SCFAs levels, significantly reducing pro-inflammatory and LPS-producing bacteria and serum LPS content, and this effect can be maintained stably until adulthood; (3) The increase of SCFAs in female offspring promotes the secretion of GLP1/2, thereby improving the intestinal barrier, insulin sensitivity and inflammation of the offspring, effectively alleviating the MS; (4) The gut microbiota may serves as a central mechanistic mediator of the transgenerational metabolic benefits conferred by maternal PHZ intervention.

## 2 Materials and methods

### 2.1 Animals and experimental design

All animal experimental procedures in this study were reviewed and approved by the Ethics Committee of the Second Clinical Medical College of Jinan University, Shenzhen People’s Hospital (approval number: AUP-220302-MXR-140-002). Female C57BL/6 mice (4 weeks) were obtained from Zhejiang Vital River Laboratory Animal Technology Co., Ltd. (Zhejiang, China; SCXK-2019-0001) and acclimated for 2 weeks under specific-pathogen-free conditions with controlled environmental parameters (temperature: 20–22 °C; humidity: 40–60%; 12-h light cycle from 8:00 to 20:00). Following the acclimatization period, 24 female C57BL/6 mice were randomly assigned to three groups (*p* = 8 per group): (1) the NCD group, received a normal control diet (NCD, 3.85 kcal/g; 10% energy from fat, 20% from protein, 70% from carbohydrates; H10010, Beijing HFK Bioscience Co., Ltd., Beijing, China) throughout the entire experimental period; (2) the HFD group, was fed a high-fat diet (HFD, 5.24 kcal/g; 20% energy from protein, 20% from carbohydrates, 60% from fat; H10060, Beijing HFK Bioscience Co., Ltd., Beijing, China) for 6 weeks before pregnancy, during pregnancy and lactation; and (3) the HFD-P group, was fed a HFD for 6 weeks before pregnancy and then received the HFD supplemented with phlorizin (PHZ, purity ≥98%, derived from apple tree root extract, Changsha Zhongren Biotechnology Co., Ltd., Changsha, China) at a concentration of 0.8 g/kg diet during the perinatal period. The ingredients and composition of the diets are listed in Supplementary Table S1. Previous studies from our laboratory have confirmed the metabolic protective effects conferred by PHZ at this dosage (25, 30).

All experimental mice were randomly assigned to their respective dietary groups, and the researchers were blinded to the group allocations during data collection and analysis to minimize potential bias. Throughout the entire study period, all mice had free access to their designated diets and sterile water ad libitum. Following a 6-week period of diet-induced (HFD) obesity, female mice (12 weeks old) were mated with age-matched control C57BL/6 J males and remained on their assigned dietary regimens throughout gestation (3 weeks) and lactation (3 weeks). During pregnancy, dams were housed individually to ensure consistent environmental conditions. At birth, litter sizes were standardized to six pups per dam to minimize nutritional disparities among litters. Given that estrogen in females exerts a metabolic protective effect (29, 31), male offspring are widely regarded as more susceptible to metabolic syndrome (9), leading to relatively limited research focus on females. To address this gap, the present study focuses exclusively on female offspring. Female pups from the three maternal diet groups were weaned at 3 weeks of age and maintained on a NCD with ad libitum feeding until 18 weeks of age. Body weight and food intake of the female offspring were monitored and recorded on a weekly basis.

**Figure 1:**
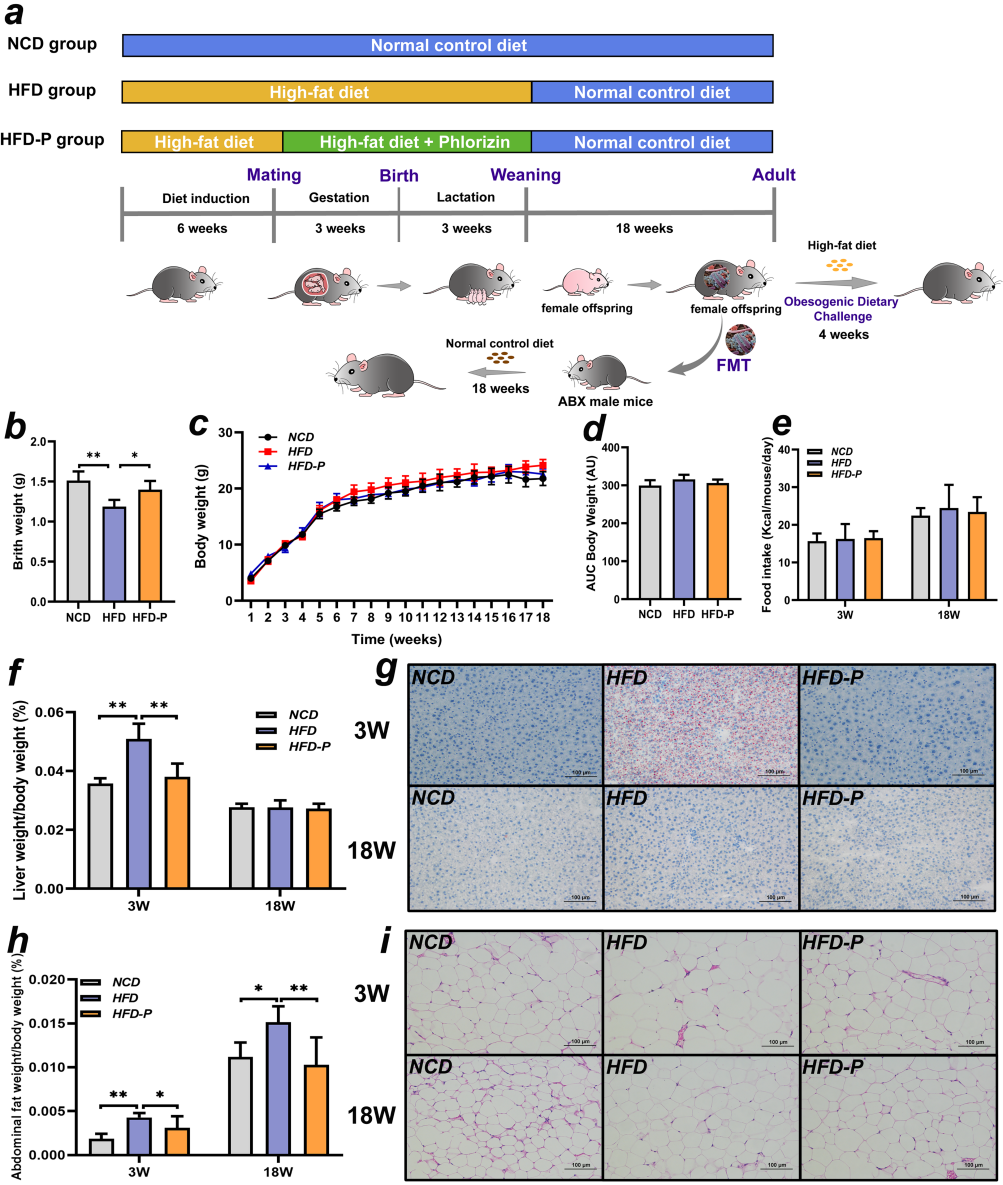
Maternal PHZ alleviated maternal HFD-induced obesity symptoms in the female offspring. **(a)** Experimental design. **(b)** Body weight, the **(c)** changes, and **(d)** AUC of body weight from birth to adulthood. **(e)** Food intake. **(f)** Liver weight relative to body weight and **(g)** Oil Red O-stained hepatic sections. **(h)** Abdominal fat relative to body weight and **(i)** H&E-stained abdominal fat sections. Bars, 100 μm. Data are presented as mean ± SD (*n* = 8). One-way ANOVA was used to analyze statistical differences; *p < 0.05, **p < 0.01, ***p < 0.001.

At 3 and 18 weeks of age, one female pup from each litter (*n* = 8 per group) was euthanized following a 12-h fast. Blood samples were collected via the intraorbital retrobulbar plexus, then centrifuged at 4,000 rpm for 10 min to isolate serum, which was subsequently stored at −80 °C. Tissue specimens including liver, ileum, and colon, along with fecal samples and abdominal fat, were preserved either at −80 °C or in 4% paraformaldehyde solution for later analysis. The liver and abdominal adipose tissues were weighed immediately after collection. All procedures were performed under chloral hydrate anesthesia, with every effort taken to reduce animal distress. The overall experimental timeline is summarized in Figure 1a.

### 2.2 Oral glucose- and insulin-tolerance test

The oral glucose tolerance test (OGTT) was performed on female offspring at 18 weeks of age. Following a 12-h fast, mice received an oral dose of glucose at 2.0 g per kg of body weight. Blood glucose concentrations were monitored using a portable glucometer (*ACCU-CHEK*^®^, Germany), with 1 μL of blood sampled from the tail vein prior to glucose administration and at 30-, 60-, 90-, and 120-min post-administration. The insulin tolerance test (ITT) was carried out in female offspring at 18 weeks of age. After a 6-h fasting period, insulin was administered intraperitoneally at a dose of 0.75 U/kg body weight. Glucose levels were measured using the same procedure as in the OGTT. The area under the curve (AUC) for both OGTT and ITT was calculated using previously methods (25).

### 2.3 Histology and immunofluorescence

Tissue samples from the liver, abdominal adipose, ileum, and colon were harvested for histopathological examination. Hematoxylin and eosin (H&E) staining, as well as Alcian blue/periodic acid-Schiff (AB/PAS) staining, were performed on tissue sections following established protocols (32). Hepatic lipid accumulation was assessed using Oil-Red O (ORO) staining on frozen liver sections, carried out as previously detailed (33). A light microscope (Eclipse Ci-L, Nikon, Japan) was employed to quantify goblet cell numbers in the stained sections.

Immunofluorescence (IF) staining was employed to evaluate mucin 2 (MUC2) expression in the ileum and colon. After deparaffinization, tissue sections were initially incubated at 4 °C overnight with a primary rabbit anti-MUC2 antibody (bsm60016R; Bioss, Beijing, China), followed by a 50-min incubation at 37 °C with a FITC-conjugated anti-rabbit secondary antibody (Bioss Inc.). Nuclei were subsequently counterstained with DAPI, and images were captured using a fluorescence microscope (Eclipse C1, Nikon, Japan).

### 2.4 Biochemical parameters analysis

Serum levels of lipopolysaccharide (LPS), total cholesterol (TC), triglycerides (TG), low-density lipoprotein cholesterol (LDL-C), high-density lipoprotein cholesterol (HDL-C), glucagon-like peptide-1 (GLP-1), GLP-2, diamine oxidase (DAO), D-lactate, and insulin, along with fecal secretory immunoglobulin A (sIgA), were quantified using ELISA kits from mlbio (Shanghai Enzyme-linked Biotechnology Co., Ltd., China), following the manufacturer’s protocols. Analyte concentrations were derived from corresponding standard curves. The homeostatic model assessment of insulin resistance (HOMA-IR) index was computed using a previously established formula (34).

### 2.5 Quantitative real-time PCR

Total RNA was isolated from colon tissue using the MiniBEST Universal RNA Extraction Kit (TaKaRa Biomedical Technology, Beijing, China) according to the manufacturer’s instructions. The purity and concentration of the extracted RNA were assessed using a Nanodrop 2000 spectrophotometer (Thermo Fisher Scientific, Waltham, MA, USA). For each sample, 1 μg of total RNA was reverse-transcribed into cDNA using the iScript cDNA Synthesis Kit (Bio-Rad Inc., USA). The qPCR was carried out on an ABI PRISM 7300 Real-Time PCR System (Applied Biosystems, Foster City, CA, USA) with SsoFast EvaGreen Supermix containing Low ROX, following the recommended protocol. Gene-specific primers are provided in Supplementary Table S2 (35, 36). GAPDH was used as the internal control, and the relative mRNA expression levels were determined using either the log2 fold change or the 2^−ΔΔCt^ method. All qPCR assays were performed in triplicate to ensure reproducibility.

### 2.6 Gut microbiota analysis

Fecal samples were collected from female offspring at 3 weeks and 18 weeks of age for 16S rDNA sequencing. Genomic DNA was extracted using the QIAamp DNA Stool Mini Kit (Qiagen, Germany) following the manufacturer’s instructions and stored at −80 °C prior to analysis. DNA concentration and integrity were assessed using a Nanodrop 2000 spectrophotometer and agarose gel electrophoresis, respectively.

The V3–V4 hypervariable regions of the bacterial 16S rRNA genes were amplified using the extracted genomic DNA as a template, with the primer pair 338F (5′-ACTCCTACGGGAGGCAGCAG-3′) and 806R (5′-GGACTACHVGGGTWTCTAAT-3′), following an established protocol (37, 38). The resulting amplicons were sequenced on the Illumina MiSeq PE300 platform (Illumina, San Diego, USA) according to the standard procedures provided by Majorbio Bio-Pharm Technology Co. Ltd. (Shanghai, China). Raw FASTQ files were de-multiplexed using an in-house perl script, and then quality-filtered by fastp version 0.19.6 and merged by FLASH version 1.2.7 with the following criteria: (i) the 300 bp reads were truncated at any site receiving an average quality score of <20 over a 50 bp sliding window, and the truncated reads shorter than 50 bp were discarded, reads containing ambiguous characters were also discarded; (ii) only overlapping sequences longer than 10 bp were assembled according to their overlapped sequence. The maximum mismatch ratio of overlap region is 0.2. Reads that could not be assembled were discarded; (iii) Samples were distinguished according to the barcode and primers, and the sequence direction was adjusted, exact barcode matching, 2 nucleotide mismatches in primer matching. Then the optimized sequences were clustered into operational taxonomic units (OTUs) using UPARSE 7.1 with 97% sequence similarity level. The most abundant sequence for each OTU was selected as a representative sequence. Sequencing data were processed using QIIME, and OTUs were generated from the sequence reads. Subsequent analyses were performed using QIIME and R software (v3.2.0). Alpha diversity metrics at the OTU level, including the Shannon, Simpson, and Chao indices, were computed based on the OTU abundance table generated in QIIME. The microbial dysbiosis index (MDI) was evaluated according to a previously published method to evaluate the degree of gut microbiota imbalance, with higher values indicating more severe microbial disruption (39). Beta diversity was assessed using Bray-Curtis metrics to examine compositional differences in microbial communities across samples, and results were visualized through principal coordinate analysis (PCoA). To identify the strains (phylum to genus) with significant differences, the linear discriminant analysis (LDA) effect size (LEfSe) approach was applied with an LDA threshold > 4 and a significance level of *p* < 0.05. Statistical comparisons of alpha diversity metrics across groups were performed using the non-parametric Kruskal–Wallis test (implemented in mothur v1.30.2). When the omnibus test indicated a significant global difference, pairwise *post-hoc* analyses were carried out with Dunn’s test. To account for multiple testing, the resulting *p*-values were corrected using the Benjamini–Hochberg false discovery rate (FDR) procedure. The significance of pairwise differences is denoted by asterisks (*) in the figures, corresponding to the following FDR-adjusted *p*-value thresholds: *p < 0.05, **p < 0.01, ***p < 0.001.

### 2.7 SCFAs analysis

Fecal short-chain fatty acids (SCFAs), including acetic, propionic, butyric, isobutyric, valeric, and isovaleric acids, were quantified using gas chromatography–mass spectrometry (GC–MS; 8890B-7000D, Agilent Technologies Inc., CA, USA). Sample preparation and the GC–MS analytical procedure were carried out following previously established protocols (40–42). Briefly, 100 mg of fecal sample was accurately weighed and transferred into a 2 mL grinding tube, followed by the addition of 500 μL of water containing 0.5% phosphoric acid. The mixture was homogenized at 50 Hz for 3 min, repeated twice, then subjected to ultrasonication for 10 min and centrifuged at 4 °C and 13,000 g for 15 min. Next, 200 μL of the resulting supernatant (fecal water) was mixed with 200 μL of n-butanol containing the internal standard 2-ethylbutyric acid (10 μg/mL) for liquid–liquid extraction. The solution was vortexed for 10 s, ultrasonicated for 10 min at 4 °C, and finally centrifuged at low temperature (4 °C) and 13,000 g for 5 min to obtain the supernatant for subsequent GC–MS analysis. The quantification of SCFAs was carried out using a HP-FFAP capillary column (30 m × 0.25 mm × 0.25 μm, Agilent Technologies, Inc.) with helium as the carrier gas at a constant flow rate of 1 mL/min. The GC oven temperature was initially held at 80 °C, then increased to 120 °C at a ramp rate of 40 °C/min, further raised to 200 °C at 5 °C/min, and finally maintained at 220 °C for 3 min. The front inlet, transfer line, and electron ionization (EI) ion source temperatures were set at 180 °C, 230 °C, and 230 °C, respectively. Data analysis was performed using MassHunter software (Version 10.0.707.0, Agilent Technologies, USA).

### 2.8 Obesogenic dietary challenge

In a separate cohort of offspring (OffNCD, OffHFD, and OffHFD-P) at 18 weeks of age, 1 female from each litter were provided *ad libitum* access to the HFD for 4 weeks, to assess the impact of an obesogenic dietary challenge on the adult phenotype.

### 2.9 Microbial depletion and fecal microbiota transplantation (FMT)

After 14 days of antibiotic treatment, the confirmation of microbiota depletion involved the utilization of BHI agar plates for fecal plating, incubated both in anaerobic and aerobic environments, along with quantitative PCR (qPCR). qPCR was performed using bacteria-specific primers (F: ACTCCTACGGGAGGCAGCAGT; R: ATTACCGCGGCTGCTGGC). To investigate whether gut microbiota plays a vital role in mediating female offspring susceptibility to obesity and to eliminate potential confounding effects of estrogen, antibiotic-treated (ABX) male mice were generated following a previously described protocol (43). Briefly, a total of 24 mice (*n* = 8 per group) were administered a cocktail of antibiotics in sterile drinking water, consisting of 1 g/L ampicillin, 1 g/L metronidazole, 1 g/L neomycin sulfate, and 0.5 g/L vancomycin hydrochloride. Antibiotic solutions and water bottles were refreshed weekly. After 14 days of treatment, the effectiveness of microbial depletion was confirmed by fecal plating on BHI agar followed by incubation under both anaerobic and aerobic conditions, as well as by qPCR using universal bacterial primers (Forward: ACTCCTACGGGAGGCAGCAGT; Reverse: ATTACCGCGGCTGCTGGC).

ABX mice received the supernatant for 7 consecutive days of oral gavage (200 μL/mouse) and were divided into three groups: NCD^FMT^ (FMT from *NCD* group), HFD^FMT^ (FMT from *HFD* group), and HFD-P^FMT^ (FMT from *HFD-P* group). FMT was conducted as previously described (44). Prior to FMT, ABX mice were allowed a two-day washout period with antibiotic-free water. For gavage preparation, fresh fecal samples were collected from 18-week-old female offspring of the three experimental groups (NCD, HFD, and HFD-P; *n* = 8 per group) and pooled within each group. Equal amounts of feces were suspended in sterile PBS at a ratio of 15 mL PBS per gram of feces, followed by vigorous vortexing for 5 min. The suspension was then centrifuged at 800 rpm for 3 min to remove large debris. The resulting supernatant was administered orally to ABX mice via gavage (200 μL per mouse) once daily for seven consecutive days. Recipient mice were assigned to three groups based on the donor source: NCD^FMT^ (receiving microbiota from the NCD group), HFD^FMT^ (from the HFD group), and HFD-P^FMT^ (from the HFD-P group). Following FMT, the ABX mice were maintained on a standard chow diet and provided sterile water freely throughout the experiment. Body weight and food consumption were monitored weekly. At 18 weeks post-FMT, OGTT and ITT were performed as previously outlined. After completion of the metabolic assessments, mice were euthanized, and blood samples, along with various organs and tissues, were collected using the procedures described earlier.

### 2.10 Data and statistical analysis

Data analysis and graphical visualization were conducted using SPSS (version 20.0; SPSS Inc., USA) and GraphPad Prism (version 8.01; San Diego, CA, USA). Before statistical analysis, the normal distribution of data was verified by the Shapiro–Wilk test, and homogeneity of variance was checked by Levene’s test. Data with normal distribution and equal variance were expressed as means ± standard deviation (SD) and compared by one-way ANOVA followed by Tukey’s *post hoc* test. Data not normally distributed were expressed as medians (interquartile range, IQR) and analyzed by the Kruskal–Wallis test. A *p* < 0.05 was considered statistically significant, with levels denoted as *p < 0.05, **p < 0.01, and ***p < 0.001. A Mantel-test based on Spearman’s correlation analysis was conducted using the VEGAN package to explore associations between metabolic parameters (SCFAs, glucolipid metabolism, inflammatory factors, intestinal permeability, intestinal function protein) and microbial community.

## 3 Results

### 3.1 Body weight and obesity-related characteristics in offspring mice

Maternal obesity or a gestational high-fat diet affects the characteristics of obesity in the offspring (45). Figure 1b showed that, the birth weight of the female offspring in HFD group significantly lower than the NCD (*p* < 0.01) and HFD-P (*p* < 0.05) group mice. In clinical practice, it has been found that low-birth-weight human infants achieve catch-up growth by consuming more nutrients and thus are more susceptible to develop metabolic syndrome in adulthood (46, 47). Subsequently, low-birth-weight female offspring of the HFD group achieved catch-up growth and did not differ significantly (*p* > 0.05) in body weight from NCD and HFD-P group mice in adulthood (Figures 1c, d). Furthermore, despite observing higher energy intake in the offspring of the HFD group at weaning (3 week) and adulthood (18 week), no statistically significant differences were found (Figure 1e).

Although there were no significant differences in body weights, the percentage of liver and abdominal fat weights in the HFD offspring group mice was significantly increased at weaning (Figures 1f,h). ORO- and H&E staining confirmed that maternal HFD promotes the hepatic fat accumulation and enlargement of fat cells in female offspring at weaning. However, these adverse metabolic effects were effectively eliminated by maternal PHZ treatment (Figure 1g). In addition, in adulthood, the female offspring of the HFD group tended to normalize their obesity symptoms, with only a slightly greater proportion of abdominal fat than in the NCD group mice (Figure 1i). In general, PHZ restores the maternal HFD-induced low-birth-weight in female offspring and improves their obesity symptoms at weaning.

### 3.2 The effects of maternal PHZ on glucolipid metabolism in offspring

**Figure 2:**
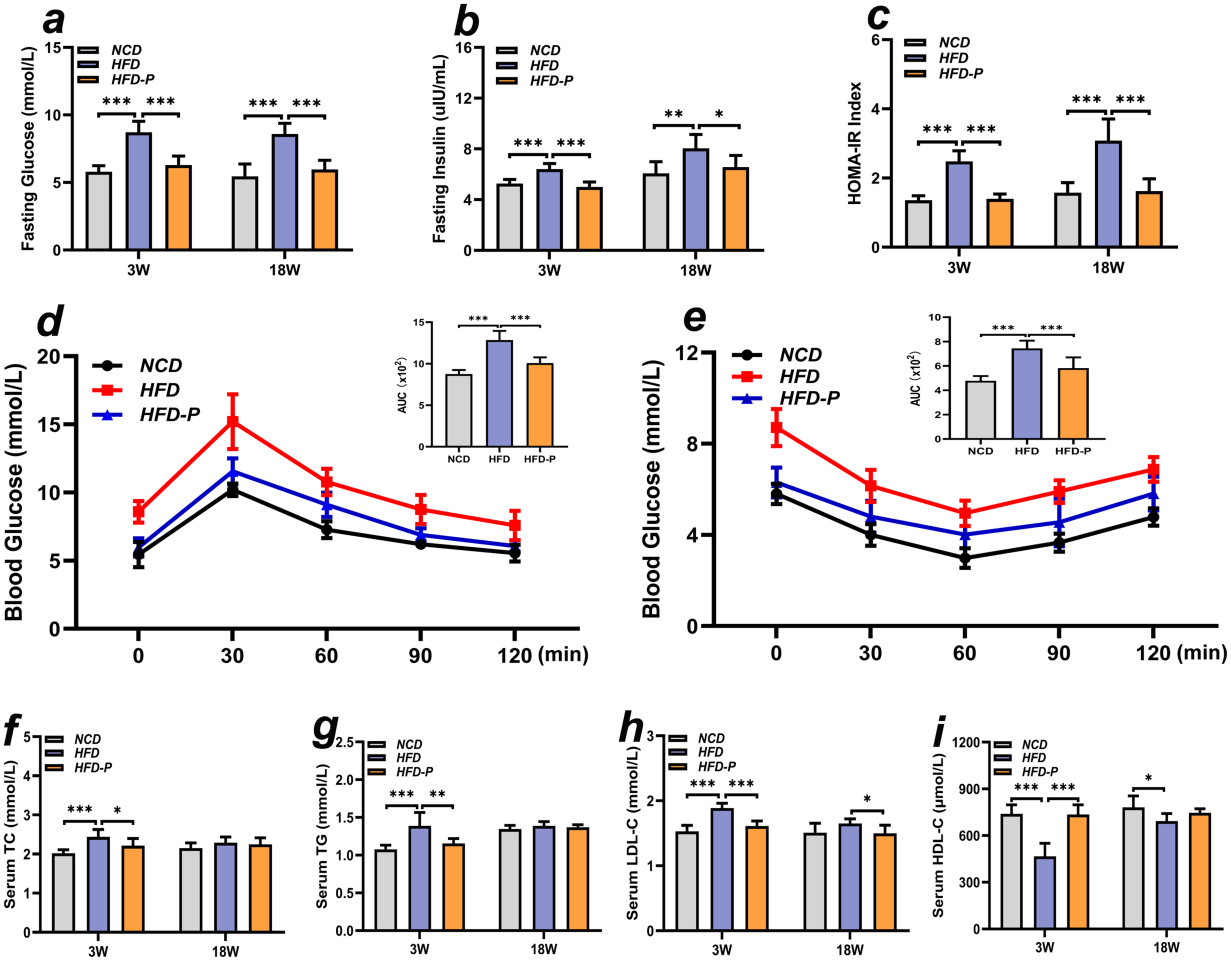
Maternal PHZ attenuated maternal HFD-induced abnormal glycolipid metabolism in female offspring. The glucose metabolism including **(a)** Fasting glucose, **(b)** Fasting insulin, **(c)** HOMA-IR index, **(d)** OGTT, and **(e)** ITT. The lipid metabolism including serum **(f)** TC, **(g)** TG, **(h)** LDL-C, and **(i)** HDL-C. Data are presented as mean ± SD (*n* = 8). One-way ANOVA was used to analyze statistical differences; *p < 0.05, **p < 0.01, ***p < 0.001.

Although there are no significant differences in body weight and obesity characteristics in adulthood. However, clinical studies have shown that low-birth-weight individuals are at higher risk of developing metabolic syndrome, regardless of the degree of obesity in adulthood. In this study, we found that female offspring from maternal HFD dams has higher fasting glucose (Figure 2a), insulin concentrations (Figure 2b) and insulin resistance levels (Figure 2c) compared to those from NCD fed dams at weaning, that these symptoms persisted into adulthood. In addition, these maternal HFD offspring mice exhibited more severely impaired glucose tolerance (Figure 2d) and lower insulin sensitivity (Figure 2e) in adulthood. However, maternal PHZ interventions improved these indices of glucose metabolism in female offspring, which the insulin tolerance levels were restored, and glucose tolerance and insulin sensitivity at adulthood were significantly improved (Figures 2a–e).

Then, the lipid metabolism in the female offspring were also evaluated. At weaning, the offspring from HFD group showed significantly higher levels of serum TC (Figure 2f), TG (Figure 2g), LDL-C (Figure 2h), and markedly reduced HDL-C level (Figure 2i) compared to those from the NCD group. Whereas, these abnormal lipid metabolisms were improved by PHZ supplementation (Figures 2f–i). Consistent with adult obesity symptoms, the abnormal lipid metabolism of HFD group female offspring in adulthood was significantly improved, including TC and TG, with only LDL-C being higher than in the HFD-P group and HDL-C being lower than in the NCD group (Figures 2f–i). Comprehensive analyses of glucolipid metabolism and insulin resistance indices confirm that maternal HFD programs key features of MS in female offspring, which are improved by maternal PHZ.

### 3.3 Maternal PHZ Attenuated HFD-induced impaired gut barrier and intestinal low-grade inflammation in adult offspring

**Figure 3:**
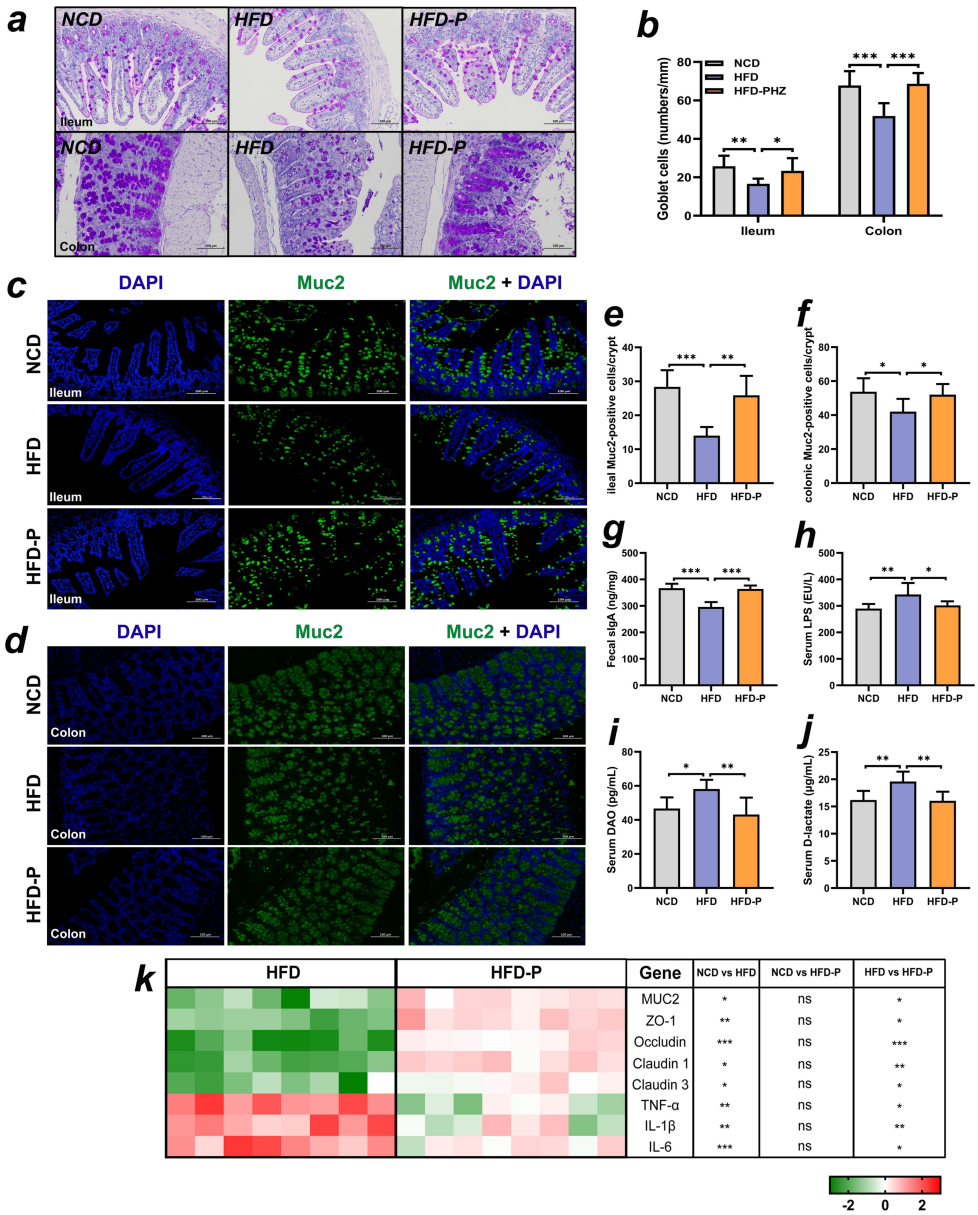
Maternal PHZ attenuated maternal HFD-induced impairment of gut barrier function in adult female offspring. **(a)** AB/PAS staining for goblet cells and **(b)** number of goblet cells. Bars, 100 μm. MUC2 expression in the **(c)** ileum and **(d)** colon was assessed by immunofluorescence, and the number of **(e)** ileal and **(f)** colonic MUC2-positive cells per crypt was quantified (*n* = 6). Bars, 100 μm. **(g)** Fecal sIgA. Intestinal permeability indicators, including serum of **(h)** LPS, **(i)** DAO, **(j)** and D-lactate. **(k)** The expression levels of genes associated with intestinal tight junctions and inflammatory responses were assessed using RT-qPCR; data are represented as log_2_ fold changes between the treatment and NCD groups. Data are presented as mean ± SD (*n* = 8). One-way ANOVA was used to analyze statistical differences; *p < 0.05, **p < 0.01, ***p < 0.001.

Increased gut permeability, metabolic endotoxemia and intestinal inflammation are important contributors to the development of insulin resistance, type 2 diabetes (T2D) and related metabolic diseases. Despite the absence of obesity symptoms and abnormal lipid metabolism in adult HFD group female offspring, severe insulin tolerance developed, revealing the presence of impaired intestinal barrier function in adulthood (Figure 2e). Furthermore, maternal obesity or high-fat-diet intake disrupts the intestinal barrier function of the offspring and causes intestinal low-grade inflammation. Therefore, we explored the effect of maternal PHZ on intestinal barrier function and inflammatory responses in female offspring in adulthood. The AB/PAS staining revealed a significant reduction in the numbers of goblet cells in both the ileum and colon of the HFD group compared to those in the NCD group. However, maternal PHZ intervention remarkably restored these cells count levels (Figures 3a,b).

MUC2 is a prominent constituent of the intestinal mucus layer secreted by goblet cells, plays a pivotal role in maintaining intestinal barrier function. The immunofluorescence staining illustrated that the ileal (Figures 3c,e) and colonic (Figures 3d,f) MUC2-positive cells were significantly decreased in the adult female offspring of the maternal HFD group. Furthermore, sIgA, an immunoglobulin presents in intestinal secretions that maintains intestinal barrier function, was also remarkably reduced in the HFD group (Figure 3g). Moreover, a series of mRNA levels related to intestinal barrier function and inflammatory response were measured (Figure 3k). The transcriptional findings indicated that the maternal HFD group offspring displayed reduced mRNA levels for MUC2, ZO-1, Occludin, and Claudin 1/3 in comparison to those from the NCD group. Furthermore, an increase in the expression of inflammatory factors TNF-*α*, IL-1β, and IL-6 was observed. With impaired intestinal barrier function in the offspring of the HFD group, we also found the serum DAO (Figure 3i) and D-lactate (Figure 3j), markers of intestinal permeability, levels in the HFD group adult female offspring were significantly elevated. In addition, there was a significant increase in serum LPS concentrations of HFD offspring, which is a key trigger for metabolic disorders such as insulin resistance and T2D (Figure 3h). However, the coadministration of PHZ effectively restored ileal and colonic MUC2 expression (Figures 3c–f) and fecal sIgA levels (Figure 3g) in adult female offspring. Also, PHZ remarkable enhanced the expression of genes related to gut barrier function (MUC2, ZO-1, Occludin, Claudin 1/3) and alleviated intestinal inflammatory response (TNF-α, IL-1*β*, and IL-6) compared to those from the HFD group (Figure 3k). Furthermore, PHZ supplementation significantly improved intestinal permeability, resulting in the normalization of abnormal serum levels of intestinal permeability markers (DAO and D-lactate) and LPS (Figures 3h–j).

These results suggest that maternal HFD led to impaired intestinal barrier function and intestinal inflammation in adult female offspring, both core pathogenic components of MS, which are ameliorated by PHZ.

### 3.4 Maternal PHZ alters gut microbiota composition and ameliorates HFD-induced gut dysbiosis in adulthood

**Figure 4:**
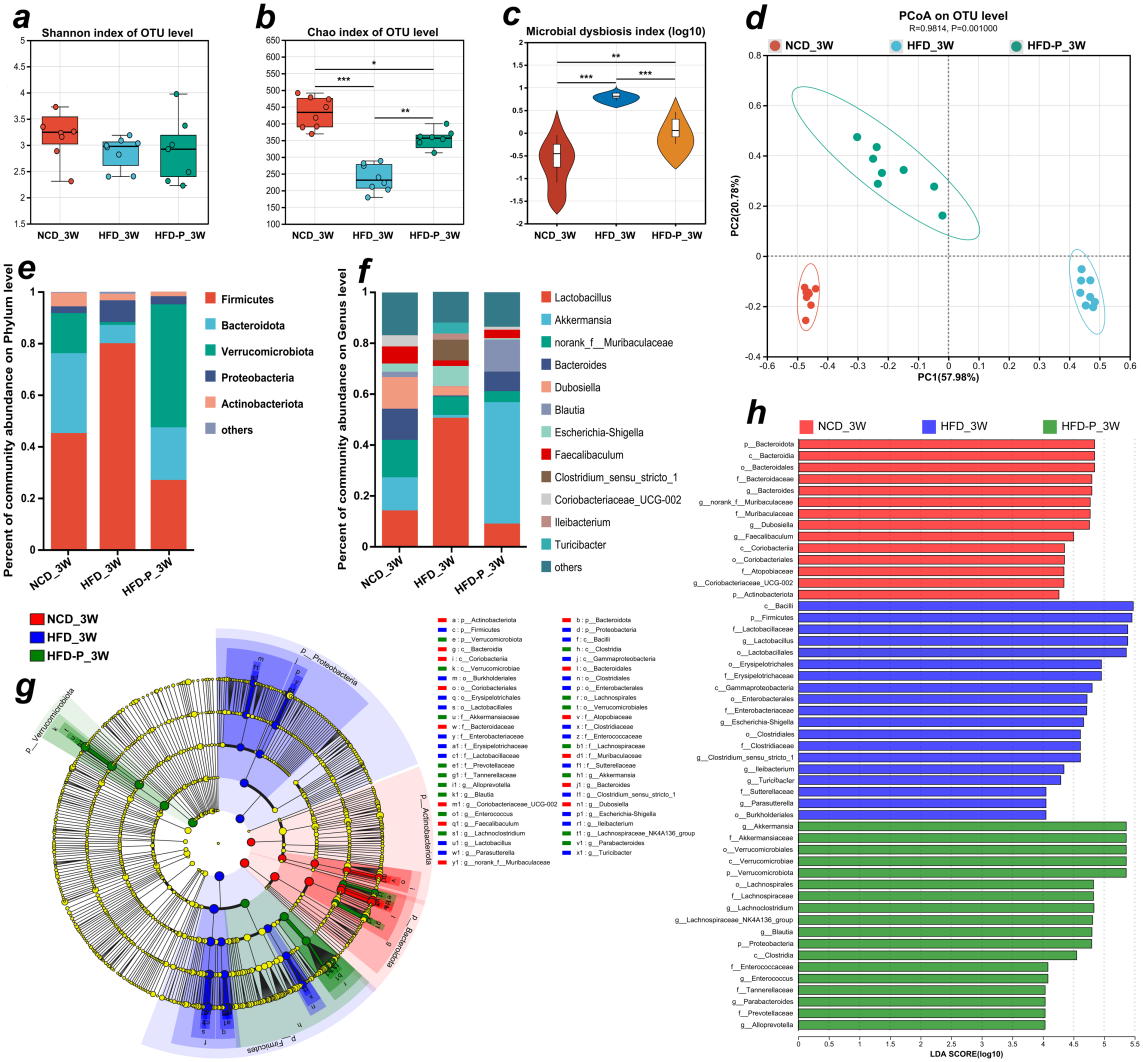
Maternal PHZ regulated the gut microbiota in female offspring at weaning (3-week-old). The *α*-diversity was evaluated through **(a)** Shannon diversity index and **(b)** Chao index. **(c)** Gut microbial dysbiosis index. **(d)** PCoA analysis comparing bacterial community variations based on the Bray-Curtis distance metric. Taxonomic composition of the gut microbiota at the **(e)** phylum and **(f)** genus levels. LEfSe analysis encompassing **(g)** LEfSe plots (*p* < 0.05; log LDA score > 4) and **(h)** LDA bar charts illustrating the microbial taxa exhibiting significant differences across the three groups, from phylum to genus level. NCD_3W, 3-week-old female offspring born to mothers fed with normal control diet; HFD_3W, 3-week-old female offspring born to mothers fed with high fat diet (HFD); HFD-P_3W, 3-week-old female offspring born to mothers fed with HFD with PHZ. Data are presented as mean ± SD (*n* = 8). One-way ANOVA was used to analyze statistical differences; *p < 0.05, **p < 0.01, ***p < 0.001.

**Figure 5:**
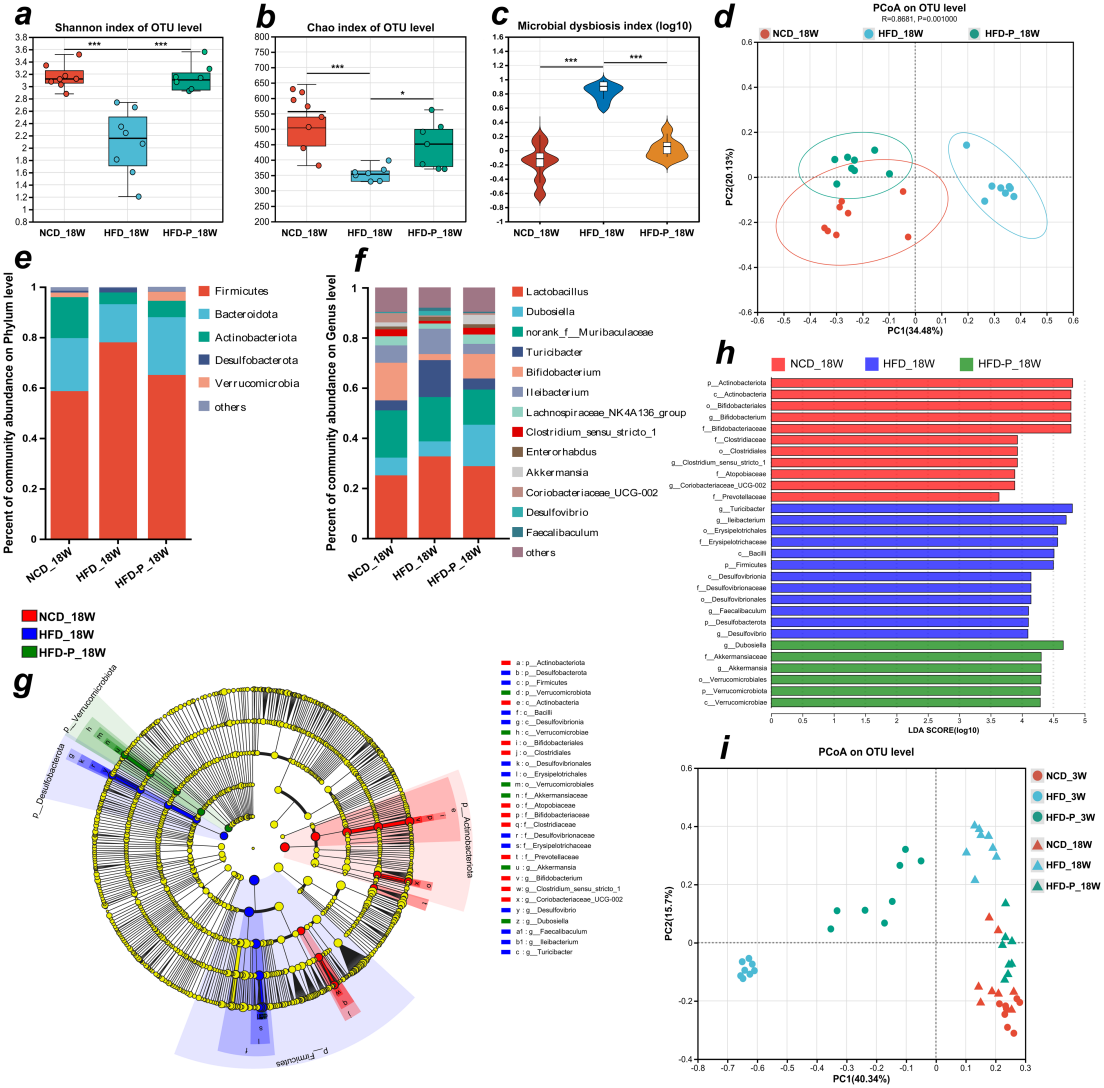
Maternal PHZ regulated the gut microbiota in female offspring at adulthood (18-week-old). The α-diversity was evaluated through **(a)** Shannon diversity index, and **(b)** Chao index. **(c)** Gut microbial dysbiosis index. **(d)** PCoA analysis comparing bacterial community variations based on the Bray-Curtis distance metric. Taxonomic composition of the gut microbiota at the **(e)** phylum and **(f)** genus levels. LEfSe analysis encompassing **(g)** LEfSe plots (*p* < 0.05; log LDA score > 4) and **(h)** LDA bar charts illustrating the microbial taxa exhibiting significant differences across the three groups, from phylum to genus level. **(i)** Overall PCoA plot comparing bacterial community shifts among the three groups in female offspring at weaning (3-week-old) and in adulthood (18-week-old). NCD_18W, 18-week-old female offspring born to mothers fed with normal control diet; HFD_18W, 18-week-old female offspring born to mothers fed with high fat diet (HFD); HFD-P_18W, 18-week-old female offspring born to mothers fed with HFD with PHZ. Data are presented as mean ± SD (*n* = 8). One-way ANOVA was used to analyze statistical differences; *p < 0.05, **p < 0.01, ***p < 0.001.

We performed 16S rDNA gene sequencing to investigate the effects of perinatal HFD and PHZ intervention on gut microbiota composition in female offspring at weaning and adulthood. At weaning, we observed that the Shannon index of female offspring was not affected by the maternal HFD or HFD-P interventions (Figure 4a). However, the Chao index was significantly lower in both the HFD and HFD-P groups compared to the NCD group, with a greater reduction in the Chao index observed in the HFD group (Figure 4b). The Shannon index reflects the species differences in different taxa (diversity), while the Chao index represents the number of different taxa in the sample (richness) (48). This indicated that although maternal HFD and HFD-P did not significantly alter the species of the offspring microbiome at weaning, they had a significant impact on its abundance. Then, the dissimilarity of β-diversity, considering species abundance, were performed by PCoA based on the Bray-Curtis distance algorithm. The plots illustrated the clear differences in microbiome between maternal HFD or HFD-P female offspring and those fed NCD at weaning (Figure 4d). The maternal NCD group was mainly concentrated in the third quadrant, while the HFD group and the HFD-P group were concentrated in the fourth and second quadrants, respectively (Figure 4d). Furthermore, we found that microbial dysbiosis index (MDI) in female offspring of maternal HFD and HFD-P groups was significantly increased compared with NCD group, and that microbial dysbiosis was more severe in HFD group (Figure 4c). The recent study revealed a significant negative correlation between MDI and the Chao index (49), which is consistent with the findings of this study. At adulthood (18 week), the *α*-diversity (Shannon and chao index) of the female progeny of HFD group was significantly reduced compared with that of NCD group (Figures 5a,b), and PCoA clustering showed that the bacterial community of HFD group and NCD group still had significant differences (Figure 5d). MDI also showed that the maternal HFD female offspring still existed serious microbial dysbiosis in adulthood (Figure 5c). However, coadministration of PHZ could normalize the α-diversity of intestinal flora in female progeny (Figures 5a,b) and significantly improve the maternal HFD-induced microbial dysbiosis (Figure 5c), as well as the microflora structure was more similar to that of NCD group (Figure 5d). In addition, the overall PCoA plot showed that although the HFD-P and NCD groups had significantly different microbiota structures at weaning, their microbiota structures were more similar in adulthood (Figure 5i). These results suggest that maternal HFD reduces the gut microbiota diversity of female progeny and causes microecological dysregulation that persists into adulthood. Although the diversity of the female offspring at weaning was significantly different after PHZ coadministration, the diversity of the female offspring’s flora and microecological structure tended to be healthy at adulthood.

Subsequently, we analyzed differences in gut microbiota composition at phyla and genus levels. At the phylum level, *Firmicutes*, *Bacteroidetes*, *Verrucomicrobiota*, *Proteobacteria*, and *Actinobacteriota* dominated the intestinal flora of female progeny at weaning (Figure 4e). While *Firmicutes*, *Bacteroidetes*, *Verrucomicrobiota*, *Desulfobacteriota* and *Actinobacteriota* dominate the microbiome in adulthood (Figure 5e). It is noteworthy that the ratio of *Firmicutes*/*Bacteroidetes* in the female offspring of the maternal HFD group is significantly increased at both weaning and adult stage. The *Firmicutes*/*Bacteroidetes* ratio of intestinal flora tends to be significantly increased in obese and diabetic individuals, which is considered as an indicator of microbiota dysbiosis (50, 51). Furthermore, the *Proteobacteria* (Figure 4e) and *Desulfobacteriota* (Figure 5e), which were enriched in the maternal HFD female offspring at weaning and adulthood, respectively, were the opportunistic pathogen or pro-inflammatory taxa, and also notably increased in the individuals with metabolic disorders (52–54). However, coadministration of PHZ can effectively diminish the levels of these detrimental strains, with the *Verrucomicrobiota* being predominantly enriched at both weaning (Figure 4e) and adulthood (Figure 5e) following PHZ intervention. At the genus level, maternal HFD significantly elevated the abundance of the *Lactobacillus*, *Escherichia-Shigella*, *Clostridium_sensu_stricto_1* and *Turicibacter* in the female offspring at weaning, and the levels of *Bacteroides*, *Blautia*, and *Akkermansia* were markedly reduced compared to those from the NCD group (Figure 4f). Conversely, PHZ coadministration significantly reduced the abundance of these HFD-enriched strains, and noticeable increased the intestinal content of *Blautia* and *Akkermansia*, which belong to the SCFA-producing bacteria and are significantly negatively correlated with obesity and T2D symptoms (Figure 4f). At the adulthood of female offspring, except for the common flora, the intestinal flora structure and abundance of NCD group and HFD-P group were similar, with *Bifidobacterium*, and *Akkermansia* being the main strains, while maternal HFD significantly enriched *Turicibacter*, and *Desulfovibrio* (Figure 5f).

Finally, the LEfSe analysis was used to finger out the key differential biomarkers in each group at various taxonomic levels. The cladogram depicts the classification of intestinal flora from phylum to genus level, with logarithmic LDA score >4.0 at weaning (3-week-old) and >3.5 at adult (18-week-old) as screening criteria, respectively. A total of 48, and 29 differential abundance taxa were identified in female offspring at weaning (Figures 4g,h) and in adulthood (Figures 5g,h), respectively. At weaning, the *Lactobacillus*, *Clostridium_sensu_stricto_1*, *Turicibacter* from *Firmicutes*, and *Escherichia-Shigella* from *Proteobacteria*, are the biomarkers for the maternal HFD group female offspring, while biomarkers for HFD-P group are *Akkermansia* from *Verrucomicrobiota* and *Blautia* from *Firmicutes* (Figures 4g,h). In adulthood, the majority of biomarkers in HFD group belonged to *Desulfovibrio* from *Desulfobacterota*, and *Ileibacterium*, *Turicibacter* from *Firmicutes*, whereas those in the HFD-P group belong to *Akkermansia* from *Verrucomicrobiota* (Figures 5g,h). These genera have been previously linked to inflammation, obesity, and metabolic disorders. However, PHZ co-intervention effectively reduced the intestinal colonization of these obesity-related bacteria. And PHZ significantly increased the intestinal levels of *Blautia* and *Akkermansia*, which are considered beneficial bacteria due to their ability to produce the SCFAs (55, 56), and they were significantly reduced in obese/T2D individuals (55, 57, 58).

Overall, maternal HFD resulted in dysregulation of intestinal microbiota in female offspring at weaning. This dysbiosis characterized by a significant increase in the abundance of bacteria associated with obesity, and it persisted into adulthood. However, PHZ co-intervention significantly improved the HFD-induced gut dysbiosis, accompanied by a significant increase in SCFA-producing bacteria in the gut and the microbiome structure in adulthood tended to be a healthy phenotype.

### 3.5 Fecal SCFAs content in adult offspring and the correlation analyses of the gut microbiota and biochemical metabolic parameters

**Figure 6:**
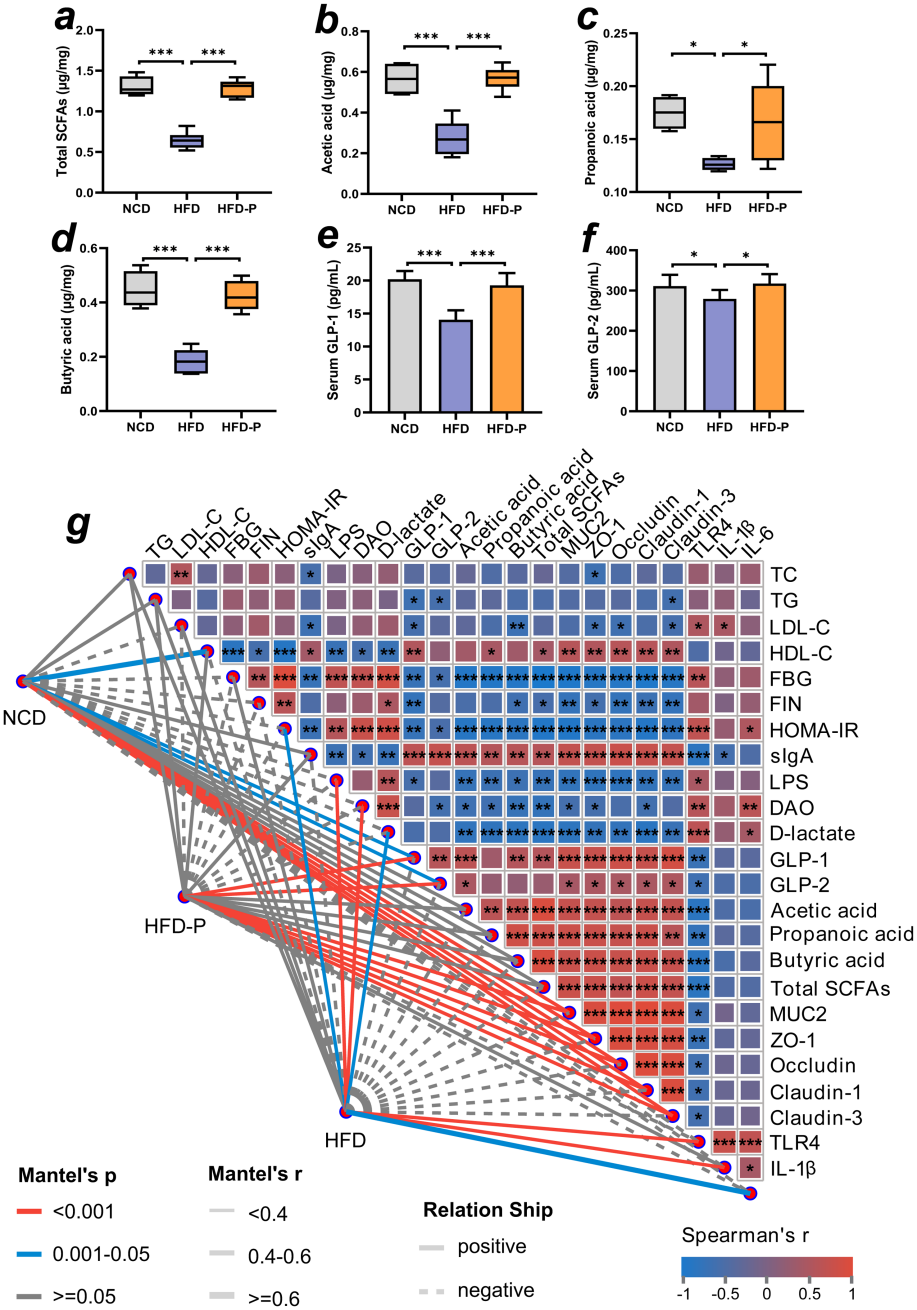
Maternal PHZ increased the fecal SCFAs and serum intestinal peptide hormones in female offspring at adulthood. Concentrations of **(a)** total SCFAs, **(b)** acetic acid, **(c)** propanoic acid, and **(d)** butyric acid in feces. Serum concentrations of **(e)** GLP-1 and **(f)** GLP-2. **(g)** Association of microbial community and metabolic parameters was analyzed by Mantel test. The edge width corresponds to the *R* value and edge color denotes the statistical significance. The color gradient indicates Pearson correlation coefficients between metabolic parameters. NCD, maternal normal control diet; HFD, maternal HFD; HFD-P, maternal HFD with PHZ. Data are presented as mean ± SD (*n* = 8). One-way ANOVA was used to analyze statistical differences; *p < 0.05, **p < 0.01, ***p < 0.001.

We quantified fecal SCFA concentrations and serum GLP-1/2 levels in adult female offspring. Additionally, the correlation between these parameters and intestinal barrier function-related/metabolism-related factors, as well as the overall composition of the intestinal microbiota in each group, was investigated. The results showed that maternal HFD significantly reduced concentrations of fecal SCFAs, especially acetic acid and butyric acid, in female offspring during adulthood (Figures 6a–d). Additionally, it significantly decreased serum levels of the peptide hormones GLP-1 and GLP-2 compared to those in the NCD group (Figures 6e,f). However, the coadministration of PHZ effectively restored the imbalances in SCFAs induced by maternal HFD, and normalized levels of intestinal peptide hormones, aligning with the observed increase in SCFA-producing microorganisms within the offspring of the HFD-P group (Figures 6a–f).

Subsequently, a Spearman correlation analysis and Mantel test analysis were performed to generate the network heat map to observe correlations between metabolic parameters and with the microbiome (Figure 6g). The results showed that SCFAs and intestinal peptide hormone (GLP-1/2) were significantly positively correlated with intestinal barrier function related indicators (MUC2, ZO-1, occludin, and claudin 1/3), while notably negatively correlated with insulin resistance (HOMA-IR), inflammatory response (TLR4), and intestinal permeability (DAO, D-lactate, and LPS). Furthermore, the microbiome of female offspring in the maternal HFD group exhibited a significant positive correlation with markers of intestinal permeability, inflammatory response, and insulin tolerance. However, the microbiome of female offspring in both the HFD-P group and the NCD group displayed a similar trend and was significantly positively associated with SCFAs, intestinal hormones (GLP-1/2), and indicators of intestinal barrier function (Figure 6g).

In summary, maternal HFD significantly reduced the levels of fecal SCFAs and serum intestinal peptide hormone (GLP1/2) in female offspring in adulthood, while PHZ co-intervention restored these abnormal metrics. At the same time, the gut microbiome of the HFD female offspring with lower levels of SCFAs and GLPs was more associated with inflammation, insulin resistance, and intestinal wall dysfunction. After PHZ co-intervention, the concentrations of SCFAs and GLPs was significantly increased, which promoted the repair of intestinal barrier function and the improvement of impaired glucose tolerance.

### 3.6 Maternal PHZ reduces susceptibility to obesity in offspring

**Figure 7:**
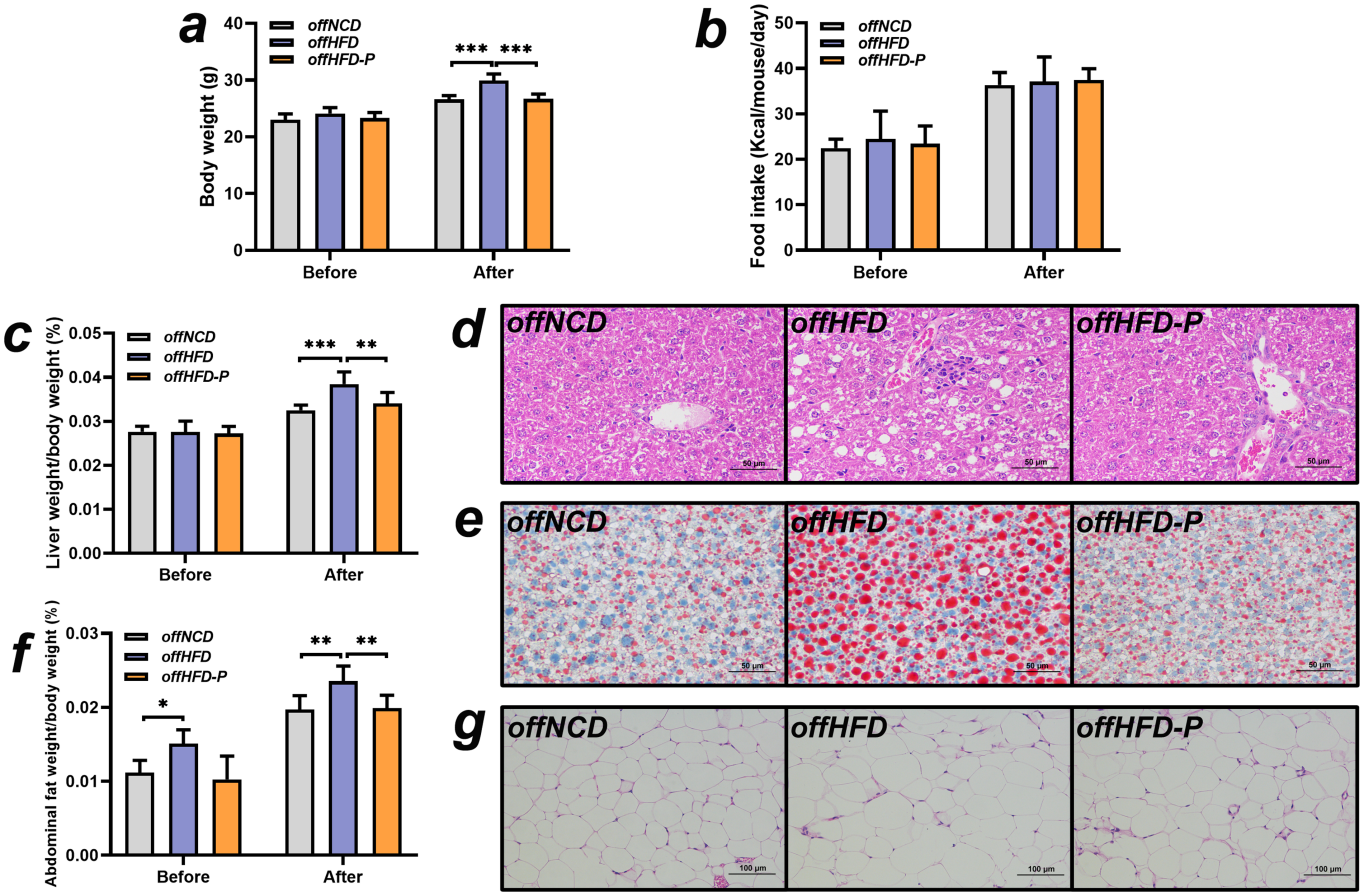
Maternal PHZ attenuates the susceptibility to obesity in adult female offspring. Female offspring at adulthood were subjected to a 4-week HFD challenge. **(a)** Body weights and **(b)** food intake. **(c)** Liver weight relative to body weight, **(d)** H&E-stained and **(e)** Oil Red O-stained hepatic sections. **(f)** Abdominal fat relative to body weight and **(g)** H&E-stained abdominal fat sections. Bars, 50 μm for hepatic sections and 100 μm for fat sections. OffNCD, adult female offspring born to maternal normal control diet; offHFD, adult female offspring born to maternal HFD; offHFD-P, adult female offspring born to maternal HFD with PHZ. Data are presented as mean ± SD (*n* = 8). One-way ANOVA was used to analyze statistical differences; *p < 0.05, **p < 0.01, ***p < 0.001.

Although the female offspring of the HFD group did not exhibit obesity symptoms in adulthood, they displayed gut microbial dysbiosis and a significant reduction in fecal SCFAs. To determine whether maternal HFD and PHZ alter obesity susceptibility in adult female offspring, we performed a 4-week high-fat diet challenge. After 4 weeks of obesogenic dietary challenges, female offspring in the maternal HFD group had a greater body weight compared to those in the NCD group. However, the addition of PHZ prevented excessive weight gain in female offspring (Figure 7a). No significant differences were found in the food intake, suggesting that the difference in weight gain was not related to food consumption or energy extraction (Figure 7b). Consistent with the significant increase in body weight, female offspring in the HFD group also exhibited a notable rise in liver weight (Figure 7c) and abdominal fat weight (Figure 7f) ratio, accompanied by more severe liver fat accumulation (Figures 7d,e) and expansion of abdominal fat (Figure 7g), as confirmed by HE and ORO staining. And these obesity symptoms were significantly improved in the female offspring through maternal PHZ co-intervention (Figures 7c–g).

These results suggest that although there is no significant difference in obesity symptoms among adult female offspring, maternal HFD progeny with dysregulated gut microbiome exhibit a significantly increased susceptibility to obesity. Furthermore, female offspring whose gut microbiota tends to normalize after PHZ co-intervention exhibit milder obesity symptoms when exposed to an obesogenic challenge compared to those in the HFD group.

### 3.7 Gut microbiota from the HFD-P adult offspring mice prevented HFD-induced impaired intestinal barrier function and obesity

**Figure 8:**
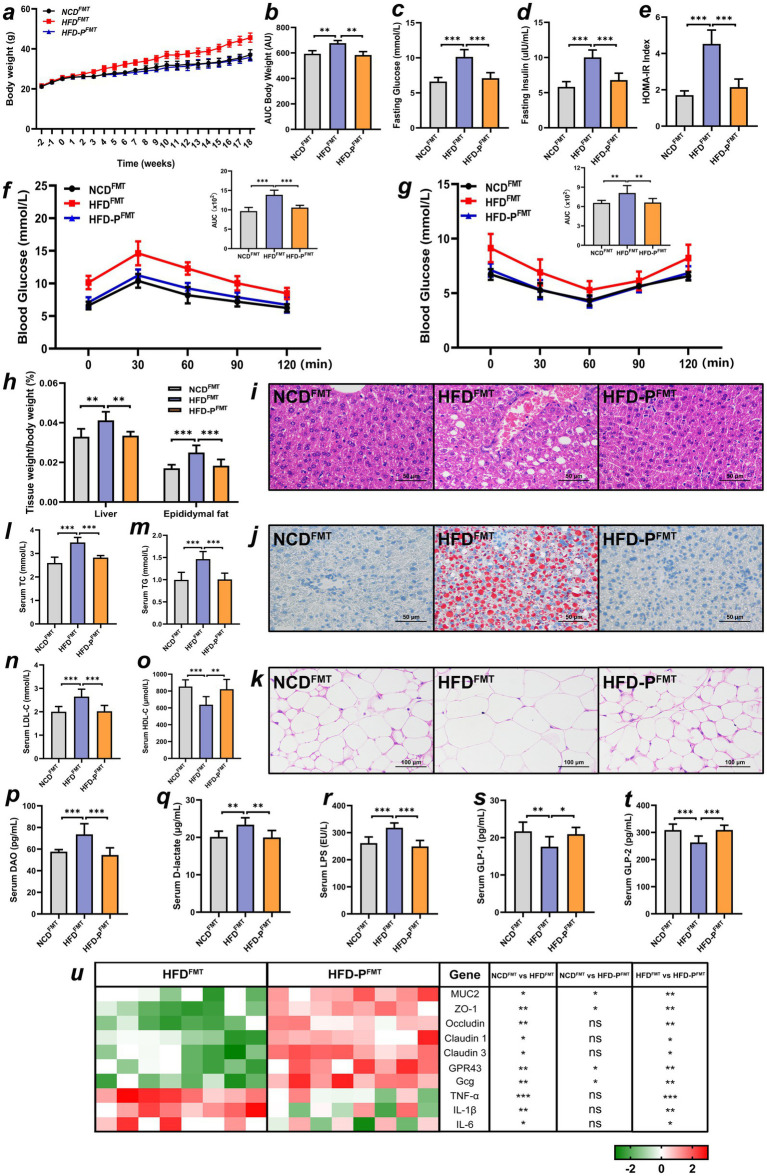
Gut microbiota mediates the transgenerational metabolic benefits of PHZ in female offspring. To exclude the metabolic protective effect of estrogen, male mice were selected as recipients for FMT. FMT from 18-week-old female offspring mice born to mothers fed PHZ ameliorated metabolic disturbances and gut barrier dysfunction induced by FMT from counterparts born to HFD-fed mothers. **(a)** Body weight and **(b)** AUC from 2 weeks before FMT to 18 weeks after FMT. Glucose metabolism includes: **(c)** Fasting glucose, **(d)** Fasting insulin, **(e)** HOMA-IR index, **(f)** OGTT, and **(g)** ITT test. **(h)** Relative tissue weight of liver and epididymis fat, and corresponding morphological staining: **(i)** H&E-stained, **(j)** Oil Red O-stained liver, and **(k)** H&E-stained epididymis fat. Lipid metabolism includes: serum levels of **(l)** TC, **(m)** TG, **(N)** LDL-C, and **(o)** HDL-C. Serum **(p)** DAO, **(q)** D-lactate, **(r)** LPS, **(s)** GLP-1, and **(t)** GLP-2 levels. And **(u)** gene expression profiles associated with gut tight junctions, inflammation, and GPR43 were analyzed by RT-qPCR, with results presented as log2 fold changes relative to the control (NCD^FMT^) group. NCD^FMT^, FMT of 18-week-old female offspring born on a maternal normal control diet; HFD^FMT^, FMT of 18-week-old female offspring born on a maternal high-fat diet (HFD); HFD-P^FMT^, FMT of 18-week-old female offspring born on a maternal HFD with PHZ. Data are presented as mean ± SD (*n* = 8). One-way ANOVA was used to analyze statistical differences; *p < 0.05, **p < 0.01, ***p < 0.001.

To exclude the metabolic protective effects of estrogen and to investigate whether the gut microbiota is a crucial factor in the difference in obesity susceptibility between female offspring, we used antibiotic-treated male mice as recipients for fecal microbiota transplantation (FMT). The ABX male mice that received FMT from adult female offspring of maternal HFD group (HFD^FMT^) exhibited significantly higher body weight under 18 weeks of normal control diet condition compared to the mice that received FMT from maternal NCD group (NCD^FMT^) (Figures 8a,b). In addition, there was a significantly impaired glucose- and insulin-resistance observed in HFD^FMT^ mice, as evidenced by notably elevation in fasting blood glucose, insulin levels, and HOMA-IR values. In addition, significantly impaired glucose tolerance (Figure 8f) and more severe insulin resistance (Figure 8g) were observed in HFD^FMT^ mice, as evidenced by a notable elevation in fasting blood glucose (Figure 8c), serum insulin (Figure 8d), and HOMA-IR values (Figure 8e). Interestingly, HFD^FMT^ mice showed severe obesity symptoms, including a greater proportion of liver weight and epididymal fat weight (Figure 8h), as well as more liver fat accumulation (Figures 8i,j) and epididymal fat cell expansion (Figure 8k). Consistent with the symptoms of obesity, HFD^FMT^ mice exhibited disturbances in lipid metabolism, including significant increases in serum TC (Figure 8l), TG (Figure 8m), LDL-C (Figure 8n) levels and significant reductions in HDL-C (Figure 8o). However, mice receiving FMT from HFD-P group (HFD-P^FMT^) alleviated HFD^FMT^-induced the symptoms of obesity and abnormal glucolipid metabolism (Figures 8a–o).

Additionally, similar to the correlation characteristics of transplanted microbiome (Figure 6g), HFD^FMT^ mice also showed a significant increase in intestinal permeability, as evidenced by a notable rise in serum concentrations of DAO (Figure 8p) and D-lactate (Figure 8q), resulting in significantly elevated levels of internal LPS (Figure 8r). At the same time, there was a notable decrease in the transcriptional expression of MUC2, ZO-1, Occludin, Claudin 1/3 gene responsible for intestinal barrier function protein in colon tissue, accompanied by an elevation in the intestinal inflammatory response (TNF-*α*, IL-1β, and IL-6) (Figure 8u). In contrast, HFD-P^FMT^ significantly improved the HFD^FMT^-induced increase in intestinal permeability (Figures 8p–r), restored the expression of genes related to intestinal barrier function, and reduced intestinal inflammation (Figure 8u). It’s worth noting that the HFD-P^FMT^ mice exhibited significantly increased levels of GLP-1 (Figure 8s) and GLP-2 (Figure 8t) compared to the HFD^FMT^ mice. This finding aligns with the considerably higher colonic expression of GPR43 and Gcg of the HFD-P^FMT^ group (Figure 8u). These results indicate that the transgenerational metabolic benefits of PHZ in female offspring are mediated through the gut microbiota-SCFA axis.

These finding indicate that gut microbiome is strongly associated with metabolic differences and predisposition to obesity in female offspring. Transplantation of PHZ-modified microbiota attenuates HFD-induced reductions in intestinal peptide hormones, disturbed glucolipid metabolism, and impaired gut barrier function, suggesting a contributory role of the gut microbiota in reducing obesity and metabolic syndrome risk in female offspring.

## 4 Discussion

Recognized by the WHO to be one of the world’s leading chronic diseases contributing to various disorders and deleterious complications, obesity is now a concerning epidemiological trend and the prevalence of which has risen startlingly over the years. By definition, obesity is a chronic, multifactorial disease characterized by excessive fat deposits that can impair physiological function and increases the risk of developing type 2 diabetes mellitus, cardiovascular disease, osteoporosis, reproductive dysfunction, sleep disturbances, and even cancers (59). Epidemiological studies have found that maternal obesity, particularly during pregnancy, predisposes offspring to an increased risk of metabolic syndrome (MS) (60–62), while maternal dietary interventions during this critical period can confer long-term beneficial effects on offspring metabolic outcomes (63). However, it remains unclear whether perinatal PHZ intake confers transgenerational metabolic benefits to female offspring. In this study, maternal HFD resulted in metabolic disorders in female offspring at weaning, including abnormalities in glucose/lipid metabolism as well as mild obesity, and these effects were alleviated by concurrent intervention with PHZ. However, in adulthood, although the female mice in the HFD group still exhibited disorders of glucose metabolism, their lipid metabolism and obesity symptoms were significantly improved. Similarly, PHZ co-intervention can improve the HFD-induced abnormal glucose metabolism in adult female offspring. This phenomenon may be attributed to the metabolic protection of estrogen in the adult female offspring. Estrogen is involved in regulating fat metabolism, including enhancing central sensitivity to leptin, upregulating insulin receptor expression in fat cells, and suppressing the lipogenic function of adipose tissue’s lipoprotein lipase activity to reduce visceral fat accumulation, thereby preventing the development of obesity in female (27, 64). Additionally, clinical studies have demonstrated that the prevalence of MS in premenopausal females is generally lower compared to males, whereas postmenopausal females exhibit a similar incidence as males (27, 28). This reveals the metabolic protective benefits of estrogen in women. Many studies have also demonstrated that adult male offspring born to mothers exposed to a high-fat or high-sugar diet often exhibit more severe symptoms of obesity compared to their female counterparts (9, 11, 29). Therefore, given by the metabolic disparities between genders, more research should be carried out in order to elucidate the imperative underlying mechanisms responsible for the metabolic benefits of maternal PHZ intervention on female offspring.

Gut microbiota plays a crucial role in maintaining intestinal health, which is closely associated with the development of MS, such as obesity and type 2 diabetes (T2D) (65). The discovery of the intrauterine microbiota and the shared microbial strains between mother and infant have revealed the existence of a vertical transmission from mother to infant (66). More importantly, it has been suggested that microbes from the gastrointestinal tract of the mother persists much longer into the offspring’s life and exerts more profound impacts on them, in comparison to those colonized in skin or vagina (67, 68). Therefore, gut microbiota might be the key to unlock the beneficial impacts of PHZ in transgenerational metabolism.

In our current study, maternal HFD significantly disrupted the composition with a reduced *α*-diversity of gut microbiota, and notably enriched the abundance of *Lactobacillus*, *Escherichia-Shigella*, *Clostridium_sensu_stricto_1* and *Turicibacter* in the female offspring at weaning. The *Lactobacillus* species are generally acknowledged as beneficial for host health; however, an excessively high proportion of *Lactobacillus* is found in the intestines of obese and T2D patients (69–72). Additionally, recent research has found that *Lactobacillus* exhibits endogenous proinflammatory properties (73–75), indicating the probability that excessive levels of *Lactobacillus* may actually promote the development of MS. *Escherichia-Shigella*, known for its role as an opportunistic pathobiont and its ability to produce LPS, is commonly found in higher abundance among individuals with obesity and diabetes, positioning it as a potential biomarker for these conditions (76). Moreover, emerging evidence indicates that *Escherichia-Shigella* is more abundant in obese women with gestational diabetes and shows a positive correlation with serum LDL and TG levels (77). *Clostridium_sensu_stricto_1* is frequently co-enriched with *Escherichia-Shigella* in obese individuals (78, 79), including in the gut microbiota of obese children (80), a finding that aligns with the results of the present study. Furthermore, evidence from a recent randomized controlled trial involving insulin supplementation demonstrated that the reduction of *Clostridium_sensu_stricto_1* was positively associated with improvements in weight and BMI, suggesting its potential role in promoting obesity development (81). Moreover, several studies have demonstrated that *Turicibacter* and *Lactobacillus* are predominant genera in obese and diabetic mouse models and exhibit significant positive correlations with MS (82, 83). These findings indicate that *Turicibacter* is closely associated with MS and elevated LPS levels, suggesting its potential role as a pro-inflammatory microorganism (84–86). Interestingly, despite being fed a normal diet during their growth period, adult female offspring from the maternal HFD group exhibited persistent dysbiosis in their intestinal microecology. Compared to female offspring from the maternal NCD group, they showed a significant reduction in *α*-diversity of the gut microbial community and a markedly distinct microbial structure. In addition to the persistent high abundance of *Turicibacter*, *Desulfovibrio* was also significantly enriched in the adult female offspring of the maternal HFD group. *Desulfovibrio* is a known LPS-producing and pro-inflammatory bacterium that commonly accumulates in the gut microbiota of individuals with obesity and diabetes (52, 81, 87). This bacterium produces LPS, which triggers intestinal inflammation by promoting the secretion of IL-6 and IL-8 from endothelial cells (88, 88). Furthermore, it possesses the metabolic capability to reduce sulfate to hydrogen sulfide (H₂S), a toxic compound that can impair the integrity of the intestinal barrier (89, 90). Notably, following combined intervention with PHZ, the abundance of these LPS-producing and pro-inflammatory bacteria was markedly reduced, concurrent with improvements in metabolic syndrome.

The enrichment of LPS-producing and pro-inflammatory bacteria in the gut of female offspring from maternal HFD groups led to higher levels of LPS in the circulation, indicating impaired intestinal barrier function in these individuals. LPS is a major component of the outer membrane of Gram-negative bacteria and can induce chronic inflammation and impair intestinal barrier function, thereby contributing to the development of MS, including obesity and insulin resistance (91, 92). On one hand, LPS compromises intestinal barrier integrity by downregulating tight junction proteins in the gut, thereby increasing intestinal permeability and promoting bacterial translocation (93, 94). On the other hand, LPS-induced disruption of intestinal barrier integrity is accompanied by intestinal dysbiosis and low-grade inflammation, which collectively impair intestinal colonization resistance and thereby increase the risk of establishment and invasion by potential pathogens in the gut (95). Furthermore, circulating LPS in the bloodstream can be recognized by immune cells and activate TLR4, thereby triggering the production of pro-inflammatory cytokines. This cascade initiates systemic chronic inflammation, which ultimately contributes to the development of insulin resistance and other metabolic syndrome phenotypes (91). In this study, we found that the serum LPS level in adult female offspring from the maternal HFD group was significantly higher than that in the control group, accompanied by increased intestinal permeability, reduced numbers of MUC2-positive and goblet cells, downregulated expression of intestinal tight junction proteins, and elevated intestinal inflammation. These results indicate that the dysregulation of the gut microbiota in adult female offspring of mothers fed a high-fat diet is associated with significantly elevated circulating LPS levels, which compromise intestinal integrity and trigger chronic inflammation. Persistent low-grade inflammation may contribute to the development of insulin resistance (91), providing a potential explanation for the presence of insulin resistance in these offspring despite the absence of overt obesity.

Interestingly, following PHZ co-intervention, the intestinal microecological imbalance in female offspring induced by maternal HFD was markedly ameliorated. This improvement was characterized by a significant restoration of microbial α-diversity, a notable reduction in the microbial dysbiosis index, and a microbiota structure in adult female offspring that more closely resembled that of the NCD group. From one perspective, PHZ significantly reduces the abundance of maternal HFD enriched LPS-producing bacteria and pro-inflammatory bacteria within the intestinal tract, lowers circulating LPS levels in the bloodstream, and consequently alleviates LPS-induced intestinal barrier dysfunction and insulin resistance. This effect may be attributed to the potent antibacterial properties of PHZ and its aglycone phloretin (PHT). In the gut, PHZ can be hydrolyzed by lactase-phlorizin hydrolase (96) and β-glucosidase (97) into its aglycone form, PHT, which demonstrates stronger antibacterial activity compared to its glycosylated precursor and exhibits significant inhibitory effects against LPS-producing pathogens such as *Porphyromonas gingivalis*, *Escherichia coli*, and *Salmonella* (98–100). From the other perspective, PHZ significantly enriched the SCFA-producing bacteria *Blautia* and *Akkermansia*, which were nearly depleted in the offspring of the HFD group. Notably, reduced abundances of these beneficial bacteria are commonly observed in individuals with obesity or T2D (101, 102). Among them, *Blautia* is reported to be a common producer of acetic acid and is widely detected in intestinal and fecal samples of mammals (56). Clinical studies have shown that the abundance of *Blautia* is significantly reduced in children with obesity or T2D compared to healthy controls (58, 103), and is negatively correlated with BMI and the risk of T2D (57, 104). It can help maintain the stability of the gut by enhancing the activity of regulatory T cells and the production of SCFAs, and prevent excessive inflammatory responses within the intestinal tract (56, 105). Furthermore, Oral administration studies have demonstrated that *Blautia* species, including *B. wexlerae* and *B. coccoides*, can alleviate HFD-induced insulin resistance and fat accumulation through the modulation of amino acid, SCFA metabolism, and gut microbiota (57, 106).

Another SCFA-producing bacterium, *Akkermansia*, is recognized as a next-generation probiotic. It enhances the production of acetic acid, propionic acid, and butyric acid in the presence of vitamin B12 (107). Depletion or reduced abundance of this symbiotic bacterium is associated with various diseases, including obesity, diabetes, hepatic steatosis, inflammation, and impaired response to cancer immunotherapy (108). *Akkermansia* can improve intestinal barrier function by significantly enhancing the expression of tight junction proteins in intestinal epithelial cells, strengthen intestinal immune defense and suppress chronic inflammation, and target host intestinal endocrine cells to promote the expression of GLP-1 and GLP-2, thereby ameliorating various metabolic disorders (108–110). Furthermore, randomized controlled clinical trials have demonstrated that *Akkermansia* can significantly improve insulin sensitivity and is safe for human use (111, 112). The *Akkermansia* can be detected in the intestines of infants during the first year of life, and its abundance gradually increases until adulthood, which may contribute to the maturation of the intestinal mucus layer and the development of the immune system ‌ (113). In this study, it was found that at the weaning stage, the PHZ intervention significantly increased the intestinal *Akkermansia* levels of female offspring, which was related to significant improvements in intestinal development, gut barrier dysfunction, and excessive inflammatory response caused by maternal HFD. In addition, our previous research demonstrated that PHZ significantly increased both *Akkermansia* abundance and SCFA levels in diabetic (*db/db*) male mice (26). In the present study, PHZ also significantly increased the intestinal abundance of *Akkermansia* and SCFA levels in the female offspring, suggesting that *Akkermansia* may be a key bacterial strain for PHZ in preventing metabolic syndrome. Therefore, the significant increase in SCFA-producing bacteria and SCFAs in the offspring following maternal PHZ intervention may play a key role in mediating the transgenerational metabolic benefits.

SCFAs are key microbial metabolites generated through the fermentation of dietary fibers by gut microbiota. As signaling molecules, SCFAs activate GPRs (GPR41/43) on intestinal endocrine cells, stimulating the release of GLP-1/2, which enhances insulin sensitivity and strengthens the gut barrier function, thereby ameliorating MS (114, 115). Growing evidence indicates that SCFAs are transferred between mother and fetus via multiple mechanisms and play a regulatory role in fetal metabolic programming, thereby conferring long-term protection against obesity and MS in offspring (116). Compared to infants, the mother represents a primary source of fetal SCFAs (117). Maternal SCFAs can enter the fetal circulation via monocarboxylate transporters (such as MCT1 and MCT4) from maternal blood, contributing to the establishment of fetal “metabolic memory” (118). Furthermore, breast milk is rich in SCFAs, particularly butyric acid, providing essential nutrients for newborns and supporting the development of a healthy gut microbiota (119). A recent study has demonstrated that SCFAs derived from maternal gut microbiota can reach the embryo via the maternal bloodstream. By activating GPR41/43 in the embryo, SCFAs promote the development of metabolic tissues, strengthen the intestinal barrier, and modulate immune regulation, thereby reducing the risk of MS in offspring (35). However, a maternal HFD significantly reduces SCFAs levels in both the intestine and breast milk, which may impair the establishment of early gut microbiota in the fetus and subsequent activation of GPRs (120, 121). Consistent with the findings of this study, fecal SCFAs in female offspring born to mothers fed HFD were significantly reduced. Concurrently, the expression of goblet cells, MUC2-positive cells, tight junction proteins, and serum GLP-1/2 in the ileum and colon was markedly decreased, with a significant positive correlation observed between these changes and the reduced in SCFAs levels. Following PHZ intervention, the gut microbial community structure was restored toward normal, the abundance of SCFA-producing bacteria was significantly increased, and SCFA levels were normalized. Moreover, the associated metabolic abnormalities were substantially ameliorated.

Although adult female offspring born to maternal HFD did not exhibit signs of obesity, their gut microbiota was disrupted, characterized by a significant increase in LPS and a marked reduction in SCFAs. Studies have demonstrated that the composition and functional activity of the gut microbiota can influence an individual’s susceptibility to obesity. Therefore, an obesogenic dietary challenge was conducted in adult female offspring mice. Offspring born to mothers with a HFD exhibited more pronounced obesity-related phenotypes. However, co-intervention with PHZ significantly attenuated these effects, indicating that the intestinal microecology is a key determinant influencing susceptibility to obesity. Consistent with this finding, other studies have demonstrated that germ-free (GF) mice receiving fecal microbiota transplantation (FMT) from offspring of obese mothers develop more severe obesity symptoms under a high-fat diet (20). Furthermore, when the gut microbiota from identical twins (one obese and one lean) were transplanted into GF mice, it was observed that recipients colonized with the microbiota from the obese twin rapidly gained weight, even when fed a normal diet (21). Moreover, under HFD conditions, mice with identical genetic backgrounds exhibit divergent phenotypes (obesity susceptibility and obesity resistance), attributed to differences in intestinal microecological composition (122). These results indicate that the gut microbiota can determine an individual’s susceptibility to obesity independently of the host’s genetic background. Therefore, although adult female offspring born to mothers fed a HFD did not exhibit overt symptoms of obesity, the induced microbial dysbiosis increased their susceptibility to MS, whereas PHZ treatment mitigated these risks.

Subsequently, we performed FMT experiments to further confirm that the gut microbiota is the key mediator of the cross-generational benefits of PHZ. To exclude the potential influence of estrogen, the FMT was performed on ABX male mice. Interestingly, ABX mice that received FMT from the maternal HFD group exhibited pronounced obesity-related phenotypes despite being maintained on a normal diet. In contrast, FMT from the PHZ-treated group significantly ameliorated these metabolic abnormalities. In particular, FMT from the PHZ treatment group significantly upregulated the expression of GPR43 and *Gcg* genes, which are known to play key roles in stimulating intestinal hormone secretion, improving glucose metabolism, and enhancing intestinal barrier function, collectively contributing to the alleviation of obesity and its associated MS (123–125). These findings suggest that the transgenerational metabolic benefits of PHZ in female offspring are mediated through the gut microbiota–SCFA axis.

## 5 Conclusion

Overall, our findings indicate that maternal HFD induces metabolic abnormalities in female offspring, characterized by dysregulated glucolipid metabolism during weaning and mild obesity. Upon reaching adulthood, although overt obesity symptoms are absent due to the protective effects of estrogen, these offspring exhibit disrupted intestinal microecology, accompanied by insulin resistance and impaired gut barrier function, leading to a significantly increased susceptibility to obesity. Maternal PHZ intervention effectively alleviates MS in female offspring induced by maternal HFD, and these benefits are associated with the gut microbiota–SCFA axis, which may contribute to enhancing gut barrier function, improving insulin sensitivity, and reducing systemic inflammation, thereby mitigating MS in female offspring. These findings indicate that PHZ may serve as a potential functional food component for preventing the transgenerational transmission of MS and interrupting the cycle of obesity.

## Data Availability

The datasets supporting the conclusions of this article are available in the NCBI Sequence Read Archive (SRA) repository under accession number PRJNA1443613, https://www.ncbi.nlm.nih.gov/bioproject/PRJNA1443613.

## Footnotes

1
https://www.healthdata.org/news-events/newsroom/news-releases/lancet-more-half-adults-and-third-children-and-adolescents

2
https://www.who.int/health-topics/obesity#tab=tab_1

## Glossary

PHZ: Phlorizin
MS: Metabolic syndrome
NCD: Normal control diet
HFD: High-fat diet
T2D: Type 2 diabetes
DOHaD: Developmental origins of health and disease
OGTT: Oral glucose tolerance test
ITT: Insulin tolerance test
AUC: Area under the curve
TC: Total cholesterol
TG: Triglycerides
LDL-C: Low-density lipoprotein cholesterol
HDL-C: High-density lipoprotein cholesterol
LPS: Lipopolysaccharide
GLP: Glucagon-like peptide
HOMA-IR: Homeostasis model assessment of insulin resistance
DAO: Diamine oxidase
sIgA: Secretory immunoglobulin A
qPCR: Quantitative PCR
GAPDH: Glyceraldehyde-3-phosphate dehydrogenase
OTUs: Operational taxonomic units
PCoA: Principal coordinates analysis
LDA: Linear discriminant analysis
LEfSe: Linear discriminant analysis of the effect size
MDI: Microbial dysbiosis index
GC–MS: Gas chromatography–mass spectrometry
SCFAs: Short-chain fatty acids
IL-1β: Interleukin 1β
IL-6: Interleukin 6
TNF-*α*: Tumor necrosis factor-α
GPR43: G-protein-coupled receptor 43
Gcg: Glucagon
ABX: Antibiotic-treated
GF: Germ-free
FMT: Fecal microbiota transplantation
