# Supplementary material for: Perinatal phlorizin alleviates maternal high-fat diet-induced metabolic syndrome in female mouse offspring and is associated with modulation of the gut microbiota
Source: Front Nutr. 2026 Jun 10;13:1799829. doi: 10.3389/fnut.2026.1799829 (PMC13290781; doi:10.3389/fnut.2026.1799829)
Supplement: Supplementary file 1 [file Table_1.docx]

**Supplementary materials**

| **Table S1.** Ingredient composition and nutrient levels of the basal diet. | | | | | |
| --- | --- | --- | --- | --- | --- |
| Product | NCD (H10010) | | | HFD (H10060) | |
|  | g | | kcal | g | kcal |
| Ingredient |  | |  |  |  |
| Casein | 189.58 | | 758.32 | 258.45 | 1033.80 |
| L-Cystine | 2.84 | | 11.36 | 3.88 | 15.52 |
| Corn Starch | 298.59 | | 1194.36 | 0 | 0 |
| Maltodextrin | 33.18 | | 132.72 | 161.53 | 646.12 |
| Sucrose | 331.77 | | 1327.08 | 88.91 | 355.64 |
| Cellulose | 47.40 | | 0 | 64.61 | 0 |
| Soybean Oil | 23.70 | | 213.3 | 32.31 | 290.79 |
| Lard | 18.96 | | 170.64 | 316.6 | 2849.40 |
| Mineral Mix M1002 | 9.48 | | 0 | 12.92 | 0 |
| Dicalcium phosphate | 12.32 | | 0 | 16.80 | 0 |
| Calcium carbonate | 5.21 | | 0 | 7.11 | 0 |
| Potassium citrate  monohydrate | 15.64 | | 0 | 21.32 | 0 |
| Vitamin Mix V1001 | 9.48 | | 37.92 | 12.92 | 51.68 |
| Choline Bitartrate | 1.90 | | 0 | 2.58 | 0 |
| FD&C yellow | 0.047 | | 0 | 0 | 0 |
| FD&C blue dye | 0 | | 0 | 0.065 | 0 |
| Total | 1000 | | 3845.70 | 1000 | 5242.95 |
|  | g% | | kcal% | g% | kcal% |
| Protein | 19.2 | | 20 | 26 | 20 |
| Carbohydrate | 67.3 | | 70 | 26 | 20 |
| Fat | 4.3 | | 10 | 35 | 60 |
| Total | — | | 100 | — | 100 |
| **Table S2.** RT-qPCR Primers Sequences. | | | | | |
| **Target gene** | **Primer** | **Sequence (5'-3')** | | | |
| *GAPDH* | Forward | GGAGAAACCTGCCAAGTATG | | | |
|  | Reverse | TGGGAGTTGCTGTTGAAGTC | | | |
| *MUC2* | Forward | TCGCCCAAGTCGACACTCA | | | |
|  | Reverse | GCAAATAGCCATAGTACAGTTACACAGC | | | |
| *ZO-1* | Forward | GGGCCATCTCAACTCCTGTA | | | |
|  | Reverse | AGAAGGGCTGACGGGTAAAT | | | |
| *Occludin* | Forward | ACTATGCGGAAAGAGTTGACAG | | | |
|  | Reverse | GTCATCCACACTCAAGGTCAG | | | |
| *Claudin 1* | Forward | GAATTCTATGACCCCTTGACCC | | | |
|  | Reverse | TGGTGTTGGGTAAGAGGTTG | | | |
| *Claudin 3* | Forward | CCTGTGGATGAACTGCGTG | | | |
|  | Reverse | GTAGTCCTTGCGGTCGTAG | | | |
| *TNF-α* | Forward | GTTCTGTCTACTGAACTTCGGG | | | |
|  | Reverse | GAGGCTTGTCACTCGAATTTTG | | | |
| *IL-1β* | Forward | ACGGACCCCAAAAGATGAAG | | | |
|  | Reverse | TTCTCCACAGCCACAATGAG | | | |
| *IL-6* | Forward | CCAGTTGCCTTCTTGGGACT | | | |
|  | Reverse | GGTCTGTTGGGAGTGGTATCC | | | |
| *GPR43* | Forward | AGGTTTGCTACTGATCCGC | | | |
|  | Reverse | GTACCCCTTCTGCTTGACTTC | | | |
| *Gcg* | Forward | TGGACTCCCGCCGTGCCCAA | | | |
|  | Reverse | CGACTTCTTCTGGGAAGTCTCGCCT | | | |
